# TLR4-mediated autophagic impairment contributes to neuropathic pain in chronic constriction injury mice

**DOI:** 10.1186/s13041-018-0354-y

**Published:** 2018-02-27

**Authors:** Yibo Piao, Do Hyeong Gwon, Dong-Wook Kang, Tae Woong Hwang, Nara Shin, Hyeok Hee Kwon, Hyo Jung Shin, Yuhua Yin, Jwa-Jin Kim, Jinpyo Hong, Hyun-Woo Kim, Yonghyun Kim, Sang Ryong Kim, Sang-Ha Oh, Dong Woon Kim

**Affiliations:** 1 Department of Plastic and Reconstructive Surgery, Department of Pediatrics, Department of Anesthesiology and Pain Medicine, Chungnam National University Hospital, Daejeon, 35015 Republic of Korea; 2 Department of Medical Science, Department of Physiology, Department of Anatomy, Brain Research Institute, Chungnam National University School of Medicine, Daejeon, 35015 Republic of Korea; 3 LES Corporation Inc., Gung-Dong 465-16, Yuseong-Gu, Daejeon, 305-335 Republic of Korea; 4 Department of Chemical and Biological Engineering, The University of Alabama, Tuscaloosa, AL 35487 USA; 5 School of Life Sciences, BK21 plus KNU Creative BioResearch Group, Institute of Life Science & Biotechnology, Kyungpook National University, Daegu, 41566 South Korea

**Keywords:** TLR4, Autophagy, Glia, Neuropathic pain, CCI

## Abstract

Neuropathic pain is a complex, chronic pain state characterized by hyperalgesia, allodynia, and spontaneous pain. Accumulating evidence has indicated that the microglial Toll-like receptor 4 (TLR4) and autophagy are implicated in neurodegenerative diseases, but their relationship and role in neuropathic pain remain unclear. In this study, we examined TLR4 and its association with autophagic activity using a chronic constriction injury (CCI)-induced neuropathic pain model in wild-type (WT) and TLR4-knockout (KO) mice. The mice were assigned into four groups: WT-Contralateral (Contra), WT-Ipsilateral (Ipsi), TLR4 KO-Contra, and TLR4 KO-Ipsi. Behavioral and mechanical allodynia tests and biochemical analysis of spinal cord tissue were conducted following CCI to the sciatic nerve. Compared with the Contra group, mechanical allodynia in both the WT- and TLR4 KO-Ipsi groups was significantly increased, and a marked decrease of allodynia was observed in the TLR4 KO-Ipsi group. Although glial cells were upregulated in the WT-Ipsi group, no significant change was observed in the TLR4 KO groups. Moreover, protein expression and immunoreactive cell regulation of autophagy (Beclin 1, p62) were significantly increased in the neurons, but not microglia, of WT-Ipsi group compared with the WT-Contra group. The level of PINK1, a marker for mitophagy was increased in the neurons of WT, but not in TLR4 KO mice. Together, these results show that TLR4-mediated p62 autophagic impairment plays an important role in the occurrence and development of neuropathic pain. And what is more, microglial TLR4-mediated microglial activation might be indirectly coupled to neuronal autophage.

## Introduction

Neuropathic pain is a complex, chronic pain state characterized by hyperalgesia, allodynia, and spontaneous pain [1]. It is caused by a lesion or dysfunction of the peripheral or central nervous system (PNS and CNS, respectively) [2]. Although it is undisputed that neurons play a fundamental role in neuropathic pain, the management of the suppression of aberrant neuronal activity has limited effectiveness and/or undesirable side effects [3]. Thus, despite progress in the development of pharmacological agents, various therapeutic agents capable of blocking abnormal pain sensation without impairing normal abilities need to be proposed.

Recently, investigations that focus on the role of the PNS immune responses after nerve injuries have highlighted the active participation of glial cells in the maintenance of chronic pain in different pathological conditions. In particular, it has been confirmed that peripheral nerve injury can induce microglia and astrocyte activation in several chronic neuropathic pain models [4, 5]. Activated microglia release various algesic substances that enhance pain transmission by neurons; particularly, proinflammatory cytokines were shown to be common mediators of allodynia and hyperalgesia [6]. Among these glial activation signals, Toll-like receptors (TLRs), particularly Toll-like receptor 4 (TLR4), have been demonstrated as initiators and mediators of neuropathic pain, [7].

TLR4 is an important pattern recognition receptor that has recently been implicated in chronic neuropathic pain [8, 9]. It recognizes pathogen-associated molecular patterns (PAMPs) and damage-associated molecular patterns (DAMPs) and regulates the innate or adaptive immune response. TLR4 has been shown to be highly expressed by microglia in the CNS of rodents [10]. Genetically altered mice with TLR4 deficiency have demonstrated significantly reduced microglia activation and pain hypersensitivity following nerve injury [7].

Autophagy is a highly regulated process involved in the turnover of long-lived proteins and damaged organelles. It involves the sequestration of regions of the cytosol within double-membrane-bound compartments and delivery of the contents to the lysosome for degradation [11]. Pain is a common feature of various neurodegenerative diseases, in which autophagy plays a critical role in the progression of the pathology and is being studied as a possible therapeutic target [12, 13]. A recent study demonstrated that autophagy is modulated differently in the spinal cord of mice in several neuropathic pain models [14]. As the most thoroughly characterized type of pattern recognition receptor, TLR4 enhances the elimination of phagocytosed mycobacteria to activate autophagy and serves as an environmental sensor for autophagy. The stimulation of TLR4 with lipopolysaccharide (LPS) induces autophagosome formation in macrophages by the TIR-domain-containing adapter-inducing interferon β (TRIF)-p38 axis and its downstream signaling pathways [15]. These results indicate that TLR4 and autophagy play a pivotal role in chronic neuropathic pain, but the mechanism remains poorly understood. Thus, in the present study, we investigated the spinal modulation of the main autophagic markers in chronic constriction injury (CCI)-induced neuropathic pain models established with wild-type and TLR4-knockout (KO) mice.

## Results

### Spinal nerve injury following CCI surgery induces mechanical allodynia in mice

**Fig. 1:**
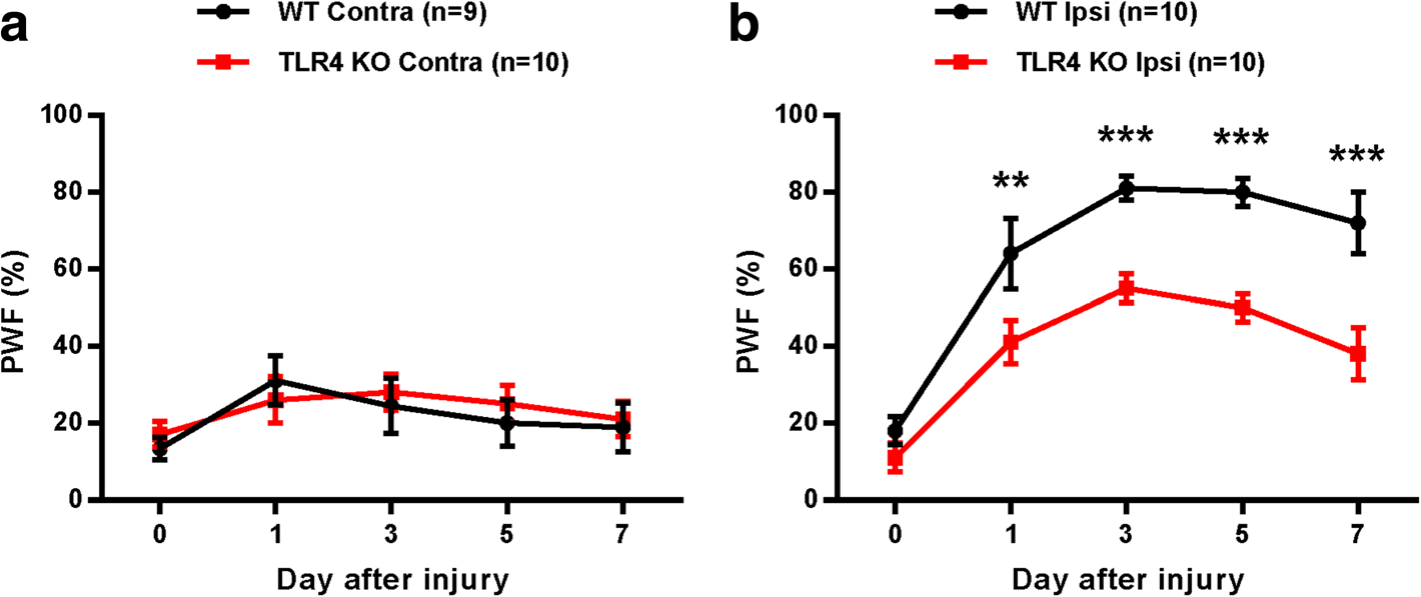
Chronic constriction injury (CCI) induces mechanical allodynia in wild-type (WT) and Toll-like receptor 4 (TLR4) knockout (KO) mice. (**a**, **b**) The paw withdrawal frequency (% PWF) was measured on days 0 (baseline), 1, 3, 5, and 7 after surgery. Mechanical allodynia was separately compared in each group of contralateral (Contra) and ipsilateral (Ipsi) in CCI mice. Two-way analysis of variance (ANOVA); all the data are shown as mean ± standard error of the mean (SEM), where *P < 0.05, **P < 0.01, ***P < 0.001, and *n* = 10 compared with the WT Ipsi group

Mechanical allodynia is a typical representation of neuropathic pain. CCI of different intensities causes an increase in mechanical allodynia [16]. To determine mechanical allodynia following CCI, we measured the hind paw PWF of mice after CCI of the sciatic nerve. In the WT group, CCI-induced mechanical allodynia caused a significant increase in PWF on the ipsilateral side compared with the contralateral side from day 1 post-surgery and was maintained for up to 7 days. A similar development of PWF on the ipsilateral side was also found in the TLR4 KO group but was significantly decreased compared with the WT group (but not on the contralateral side) in the days after surgery (Fig. 1).

### Different nociceptive effects between WT and TLR4 KO mice on CCI-induced CatWalk analysis

**Fig. 2:**
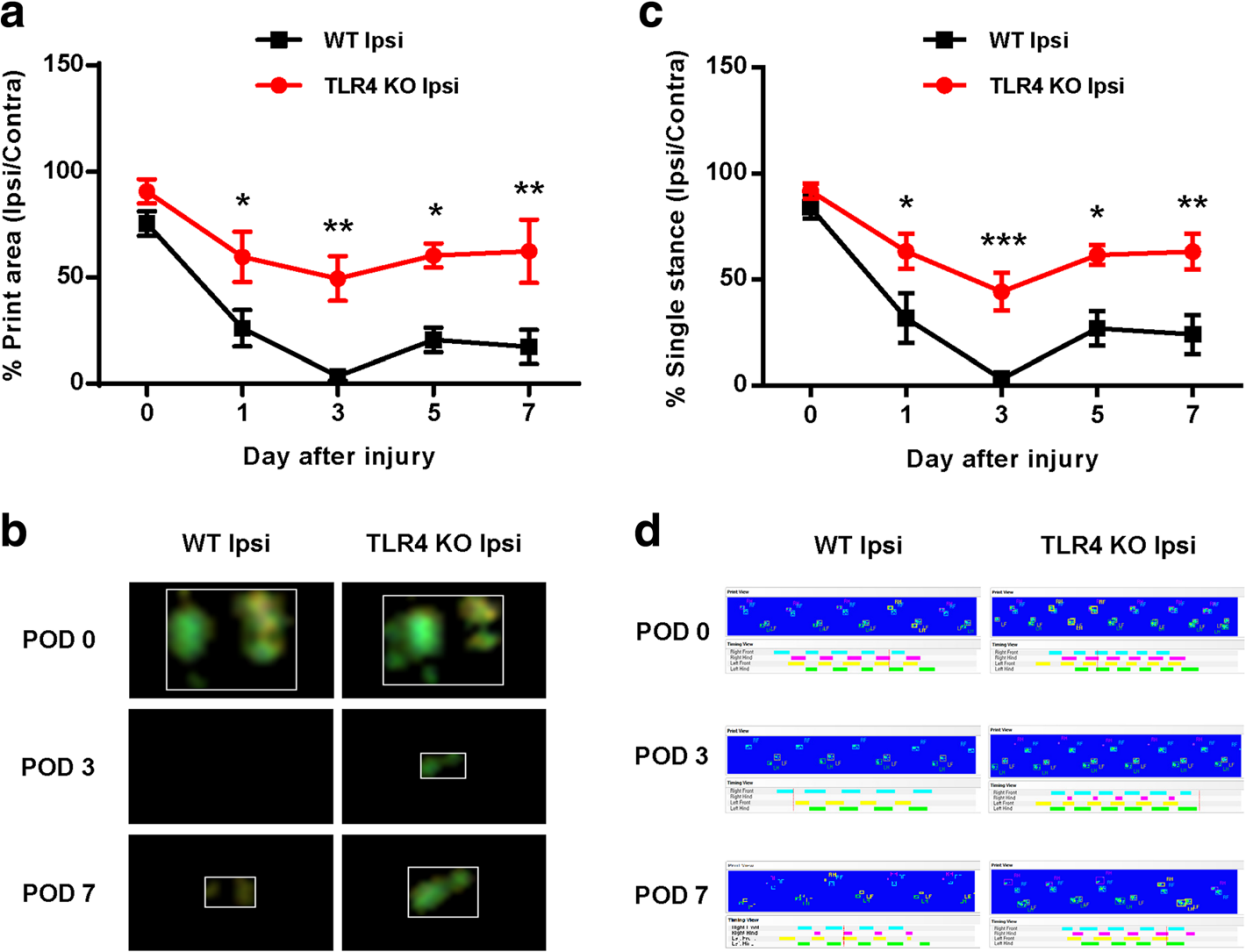
Walking track analysis in CCI mice. **a** Percentage of the Ipsi paw print area (% ipsilateral/total area) assessed in the CatWalk analysis. The paw print area (%) was increased in TLR4 KO mice. **b** Combined paw print image. **c** Percentage of the Ipsi paw single stance (% Ipsi/Contra single stance). **d** Representative digitized paw prints and associated step cycles. Two-way ANOVA; all the data are shown as mean ± SEM, where *P < 0.05, **P < 0.01, and n = 10 compared with the WT Ipsi group

CatWalk gait analysis has been used to assess gait variation and is recommended as an objective method to evaluate sensory neuropathy induced by CCI surgery [16, 17]. In the present study, we measured two parameters that were altered significantly in the ipsilateral hind paw of CCI mice: the print area was measured by calculating the surface area of the complete print of the hind paw, and the single stance was measured using the duration of the single hind paw touching the glass plate. The percentage of the print area and single stance (% ipsilateral/contralateral) was almost 100% before CCI surgery in either WT or TLR4 KO mice. However, in the WT group, the percentage was decreased almost to 0% on day 3 after CCI surgery and increased to 20% on subsequent days (Fig. 2a and c). The decreased percentage in the TLR4 KO mouse groups was observed, but reduced 50% on day 3 after surgery (Fig. 2a and c). The typical graph demonstrated that the print area and single stance disappeared in CCI mice because the mice did not step on the glass plate (Fig. 2b and d).

### Activation of microglia in the spinal dorsal horn following CCI

**Fig. 3:**
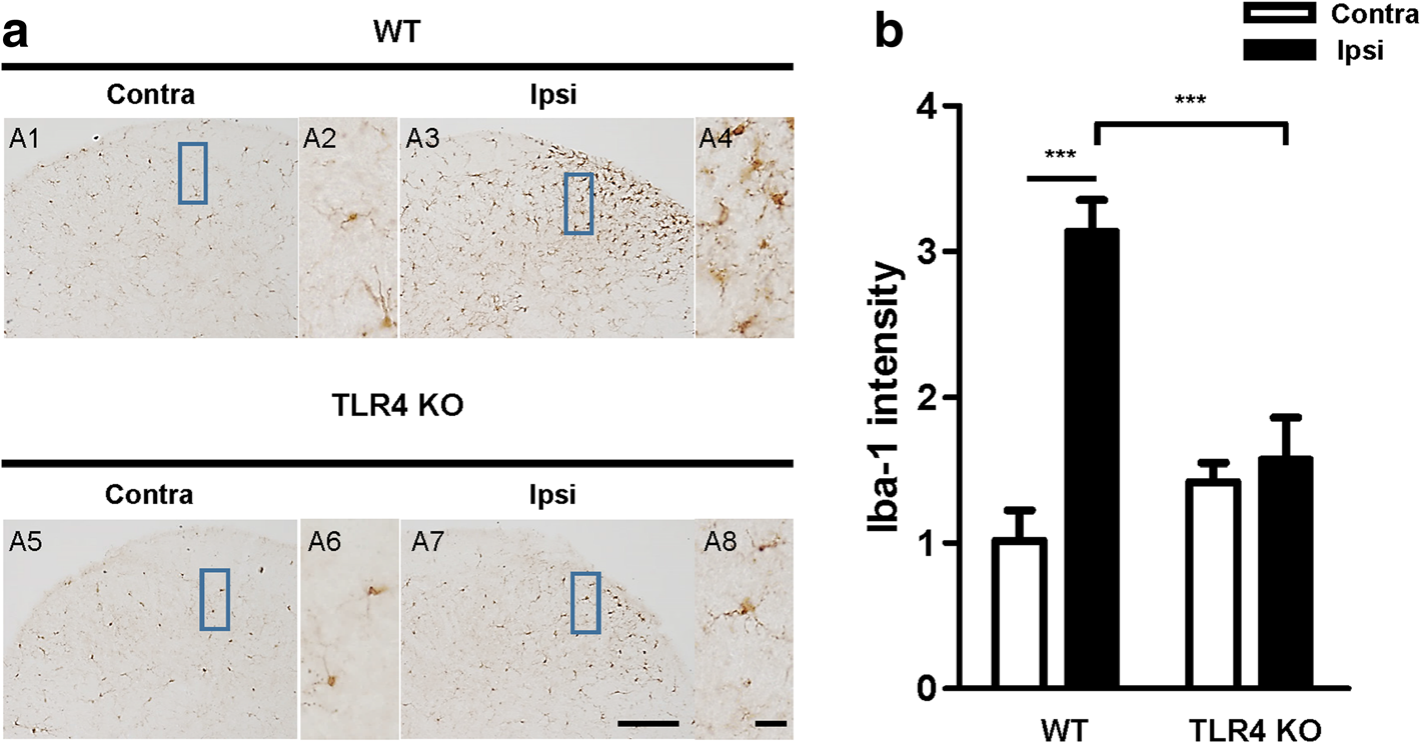
Expression of microglial in the spinal dorsal horn in WT and TLR4 KO mice. **a** The expression of microglia in the spinal dorsal horn was measured by immunohistochemistry (IHC) with Iba1 antibody. The number of microglia were significantly higher in WT Ipsi (A3, A4) superficial laminae of the dorsal horn than in the contralateral group (A1, A2). No significant increase in Iba1-immunoreactive (IR) cells was assessed in TLR4 KO mice. Scale bar = 50 μm in A1, A3, A5, and A7. Scale bar = 20 μm in A2, A4, A6, and A8. **b** The density of microglia in the superficial dorsal horn of mice was quantified with ImageJ. Two-way ANOVA; all the data are shown as mean ± SEM, where ***P < 0.001 denotes a significant difference compared with the control group

Recent studies have indicated a critical role of spinal cord microglia in the genesis of neuropathic pain [16, 18, 19]. To demonstrate the induction of neuropathic pain in our CCI model, microglia activation in the spinal cord was examined by immunohistochemical analysis with the microglia marker Iba1. CCI induced the upregulation of Iba1 in the ipsilateral spinal cord of WT mice, especially in the lamina 1–2 of the dorsal horn; however, in the contralateral spinal cord, few Iba1-immunoreactive (IR) cells could be detected (Fig. 3a). Compared with the WT mice, there was no significant increase in IR cells in the ipsilateral spinal cord compared with the contralateral spinal cord in TLR4 KO mice (Fig. 3b).

### Activation of astrocytes in the spinal dorsal horn following CCI

**Fig. 4:**
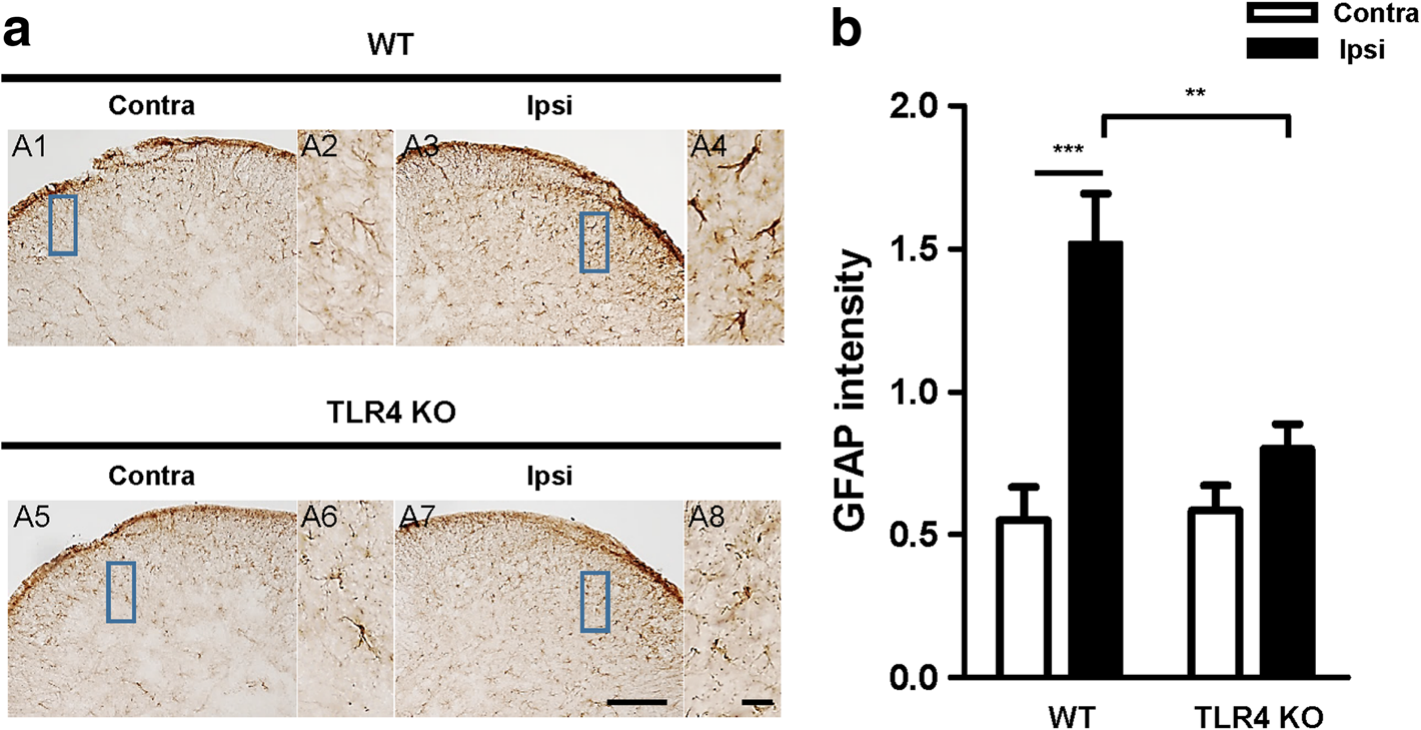
Expression of astrocytes in the spinal dorsal horn in WT and TLR4 KO mice. **a** Expression of astrocytes in the spinal dorsal horn was measured by IHC with the glial fibrillary acidic protein (GFAP) antibody. The upregulation of astrocytes was significantly higher in WT ipsilateral (A3) superficial laminae of the dorsal horn compared with the contralateral group (A1). No significant increase in IR cells was assessed in TLR4 KO mice. Scale bar = 50 μm in A1, A3, A5, and A7. Scale bar = 20 μm in A2, A4, A6, and A8. **b** The density of microglia in the superficial dorsal horn of mice was quantified with ImageJ. Two-way ANOVA; all the data are shown as mean ± SEM, where ***P < 0.001 denotes a significant difference compared with the control group

Astrocyte and microglia have different effects on neuronal activity; however, they share some common functions and are both activated in neuropathic pain [20, 21]. Therefore, we measured astrocyte activation in the same way as microglia. Similar to microglia, the upregulation of astrocytes was significantly increased in the superficial lumbar dorsal horn of the ipsilateral spinal cord compared with the contralateral spinal cord in WT mice, while no significant difference was found in TLR4 KO mice (Fig. 4).

### LC3 levels in the spinal dorsal horn following CCI

**Fig. 5:**
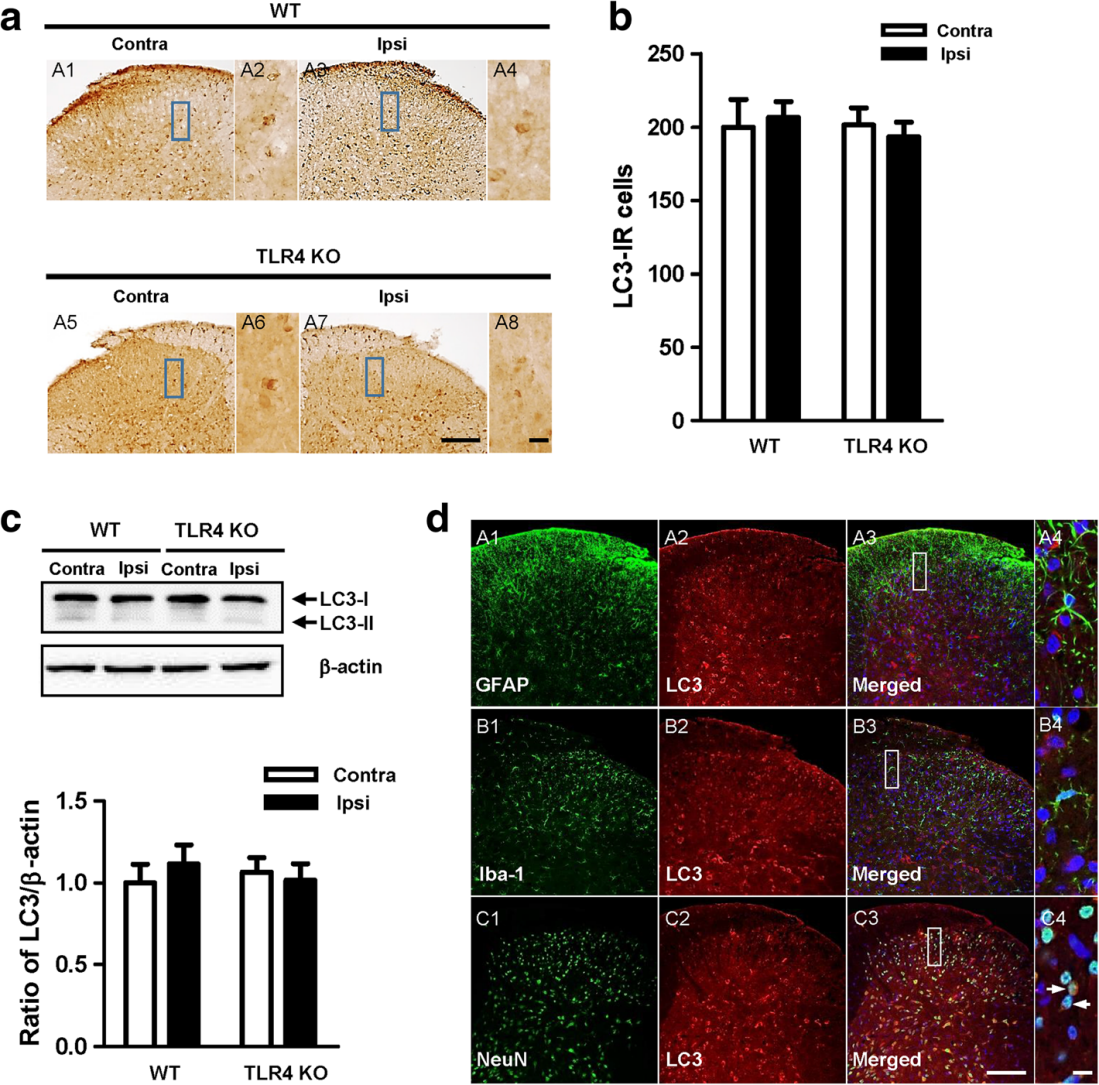
Regulation of the autophagic marker microtubule-associated protein 1 light chain 3 (LC3) in WT and TLR4 KO mice. **a** LC3 immunoreactivity can be observed in the spinal dorsal horn of CCI mice. Compared with the contralateral side, no significant increase was shown in the ipsilateral side. Scale bar = 50 μm in A1, A3, A5, and A7. Scale bar = 20 μm in A2, A4, A6, and A8. **b** The number of LC3 IR cells showed no difference in WT and TLR4 KO mice in the superficial dorsal horn. **c** The protein levels of LC3-I and LC3-II were detected with immunoblotting. Levels of β-actin were used as the loading control. Western blot analysis revealed that the levels of LC3 showed no difference in CCI mice. The band densities were analyzed with ImageJ and are expressed as a percentage of the control. The bars indicate mean ± SEM. **d** Frozen sections (WT-CCI, POD7) were stained with LC3 and co-stained with anti-GFAP (A1–4), anti-iba-1 (B1–4), and anti-NeuN antibodies (C1–4). A4, B4 and C4 is rectangular magnification of merged A3, B3 and C3, respectively. Scale bar = 50 μm in A, B, C1–3. Scale bar = 20 μm in A, B, C4

Microtubule-associated protein 1 light chain 3 (LC3) was the first mammalian protein demonstrated to be specifically associated with autophagosomal membranes [22]. It has two forms—non-lipidated and lipidated—known as LC3-I and LC3-II, respectively. LC3-II plays an essential role during the expansion step of autophagosome formation and is regarded as the most representative marker of macroautophagy [23]. The expression of LC3 was examined by immunohistochemical analysis in the spinal dorsal horn 7 days after CCI. CCI induced no significant upregulation of LC3 between the ipsilateral and contralateral spinal dorsal horn in either WT or TLR4 KO mice (Fig. 5a and b). Similarly, Western blot analysis exhibited no statistically significant variation in LC3-II expression between the ipsilateral and contralateral sides of the dorsal horn in both WT and TLR4 KO mice (Fig. 5c). Double immunofluorescence staining to detect the cellular localization of LC3 showed LC3 was expression in neuronal cells, not astrocyte and microglia in spinal dorsal horn (Fig. 5d).

### Beclin 1 levels in the spinal dorsal horn following CCI

**Fig. 6:**
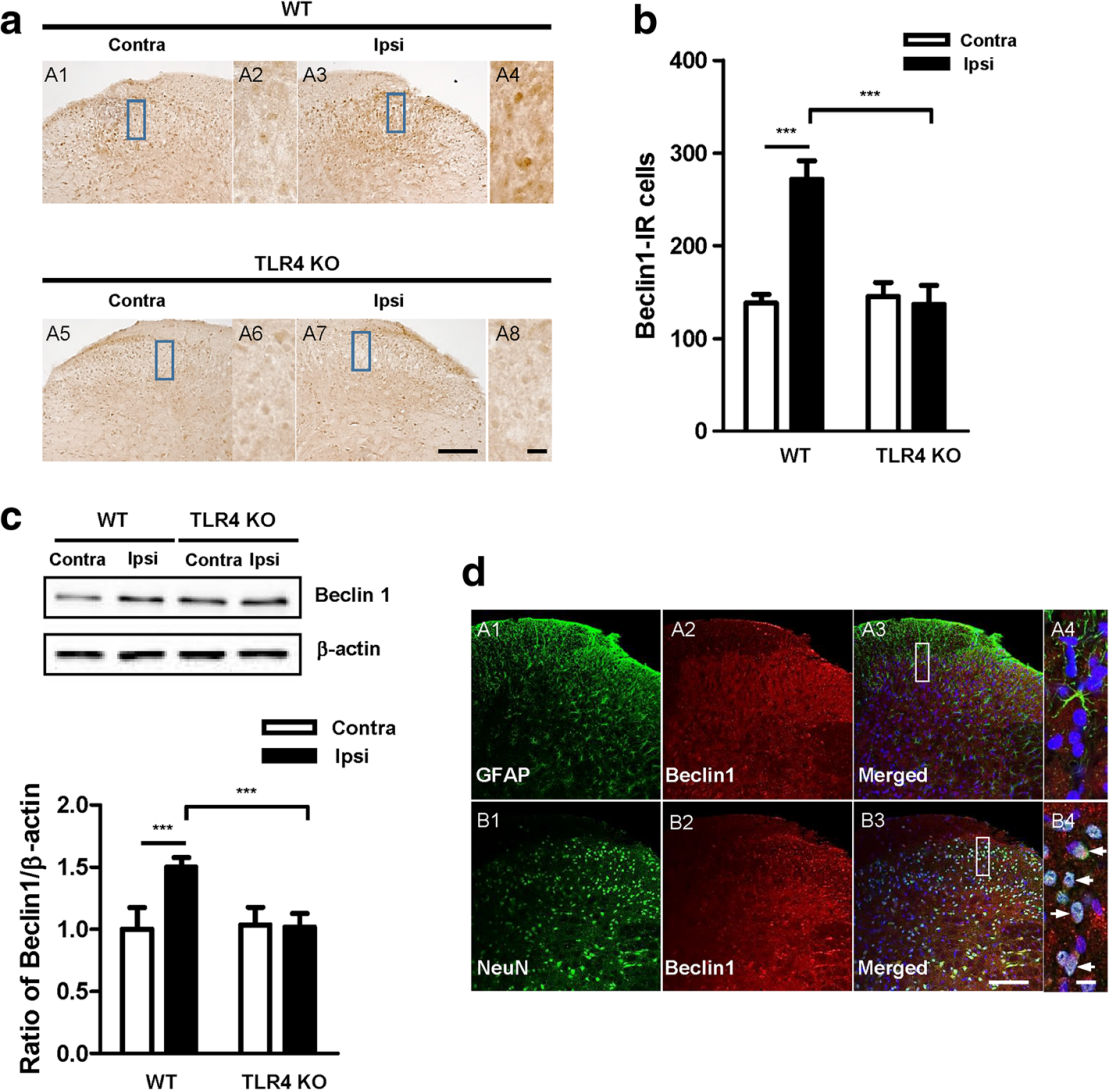
CCI induces a change in Beclin1 modulation in WT and TLR4 mice. **a** Beclin1 immunoreactivity was observed in the spinal dorsal horn of CCI mice. Compared with the contralateral side, a significant increase was shown in the ipsilateral side of WT mice, and no significant difference was found in TLR4 KO mice. Scale bar = 50 μm in A1, A3, A5, and A7. Scale bar = 20 μm in A2, A4, A6, and A8. **b** The number of Beclin 1 IR cells was significantly increased in the ipsilateral side compared with the contralateral side of WT mice. **c** The protein levels of Beclin1 were detected with immunoblotting. Western blot analysis indicated an increase in the levels of Beclin1 in the ipsilateral side compared with the contralateral side of WT mice. The band densities were analyzed with Image J and expressed as a percentage of the control. Two-way ANOVA; all the data are shown as mean ± SEM, where *P < 0.05 denotes a significant difference compared with the control group. **d** Frozen sections (WT-CCI, POD7) were stained with Beclin1 and co-stained with anti-GFAP (A1–4) anti-NeuN antibodies (B1–4). A4 and B4 is rectangular magnification of merged A3 and B3, respectively. Scale bar = 50 μm in A, B1–3. Scale bar = 20 μm in A, B4

Activation of autophagy does not only depend on LC3 but also on a series of related proteins coordinated in various steps from initiation to degradation. Beclin 1 is one of the protein markers of autophagy that plays an essential role in the induction and formation of autophagosomes [24]. Thus, we measured the expression of Beclin 1 in the spinal cord of mice by immunohistochemical and Western blot analyses. CCI induced the upregulation of Beclin 1 in the ipsilateral spinal dorsal horn in WT mice, particularly in the lamina 1–2 of the dorsal horn. Beclin 1-immunoreactive cells were increased significantly in the ipsilateral compared with the contralateral spinal dorsal horn. There was no upregulation of Beclin 1 in the spinal dorsal horn of TLR4 KO mice, and there were no significant differences between the ipsilateral and contralateral dorsal horn (Fig. 6a and b). Western blot analysis indicated a significant increase in Beclin 1 expression in the ipsilateral compared with the contralateral spinal cord in WT mice. In contrast, no significant difference in Beclin 1 expression was observed in TLR4 KO mice (Fig. 6c). Double immunofluorescence staining to detect the cellular localization of Beclin 1 showed that Beclin 1 was expressed in neuronal cells, not astrocyte in spinal dorsal horn (Fig. 6d).

### p62 levels in the spinal dorsal horn following CCI

**Fig. 7:**
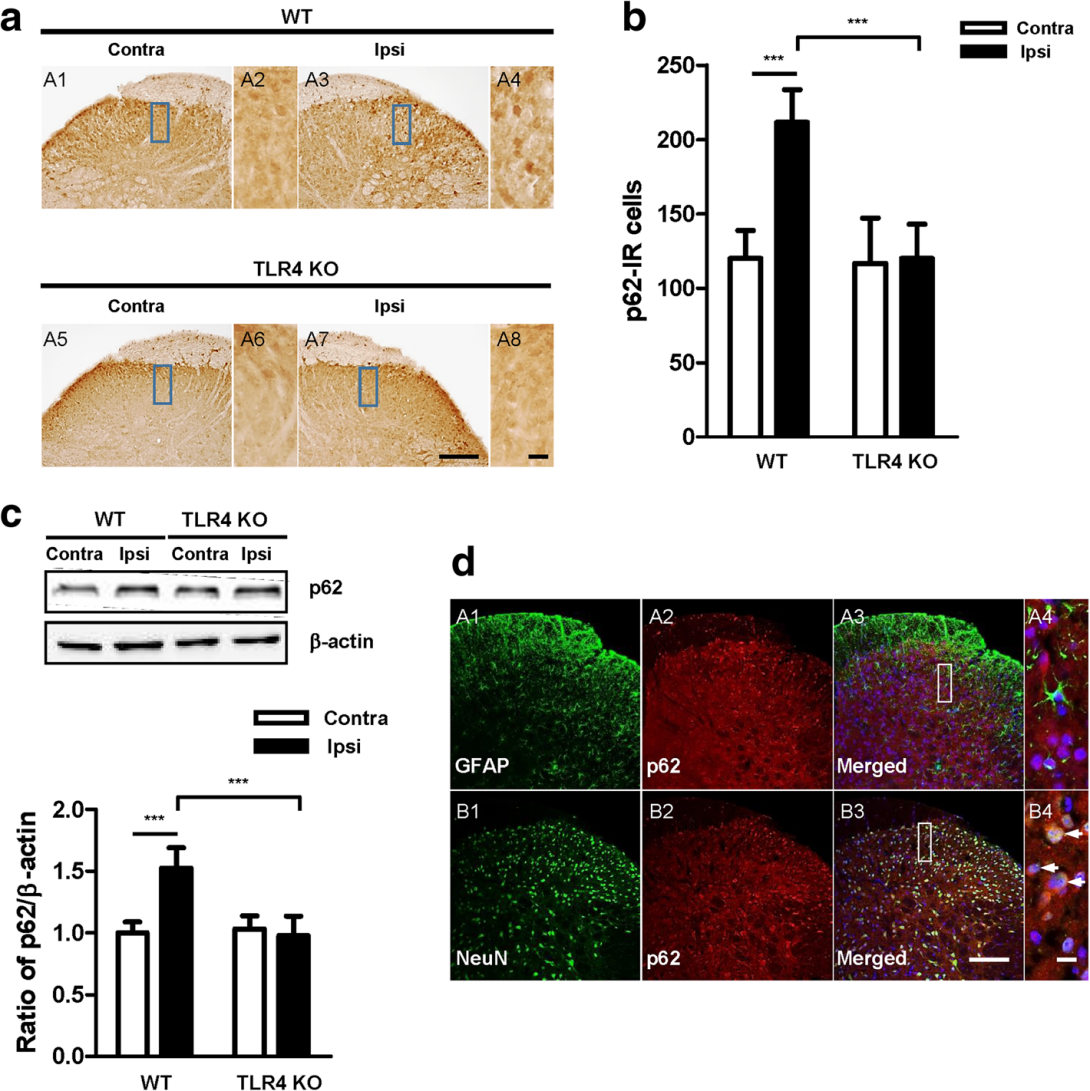
Activation of p62 in WT and TLR4 mice. **a** p62 immunoreactivity was observed in the spinal dorsal horn of CCI mice. Compared with the contralateral side, a significant increase in p62 IR cells was shown in the ipsilateral side of WT mice, and no significant difference was found in TLR4 KO mice. Scale bar = 50 μm in A1, A3, A5, and A7. Scale bar = 20 μm in A2, A4, A6, and A8. **b** The number of p62 IR cells was significantly increased in the ipsilateral side compared with the contralateral of WT mice. **c** Western blotting was performed to measure the protein expression of p62. Western blot analysis indicated an increase in the levels of p62 in the ipsilateral compared with the contralateral side of WT mice. The corresponding densitometric analysis is shown as bar graphs using Image J. Two-way ANOVA; all the data are shown as mean ± SEM, where *P < 0.05 denotes a significant difference compared with the control group. **d** Frozen sections (WT-CCI, POD7) were stained with p62 and co-stained with anti-GFAP (A1–4) and anti-NeuN antibodies (B1–4). A4 and B4 is rectangular magnification of merged A3 and B3, respectively. Scale bar = 50 μm in A, B1–3. Scale bar = 20 μm in A, B4

As one of the well-known autophagy substrates, p62/SQSTM1 is widely used to monitor autophagic flux [25]. p62 binds to LC3 directly in autophagosomes and is degraded in functional autolysosomes [26, 27]. Therefore, p62 serves as a target marker to analyze autophagic degradation. Here, we evaluated p62 expression by immunohistochemical and Western blot analyses in the spinal dorsal horn of mice. In WT mice, p62 was upregulated significantly in the ipsilateral side of the spinal dorsal horn compared with the contralateral side, but no significant change was observed in TLR4 KO mice (Fig. 7a and b). Western blot analysis exhibited the same pattern as the immunohistochemical analysis (Fig. 7c). Double immunofluorescence staining also showed that p62 was expression in neuronal cells, not astrocyte in spinal dorsal horn (Fig. 7d).

### Expression of PINK1 in the spinal dorsal horn following CCI

**Fig. 8:**
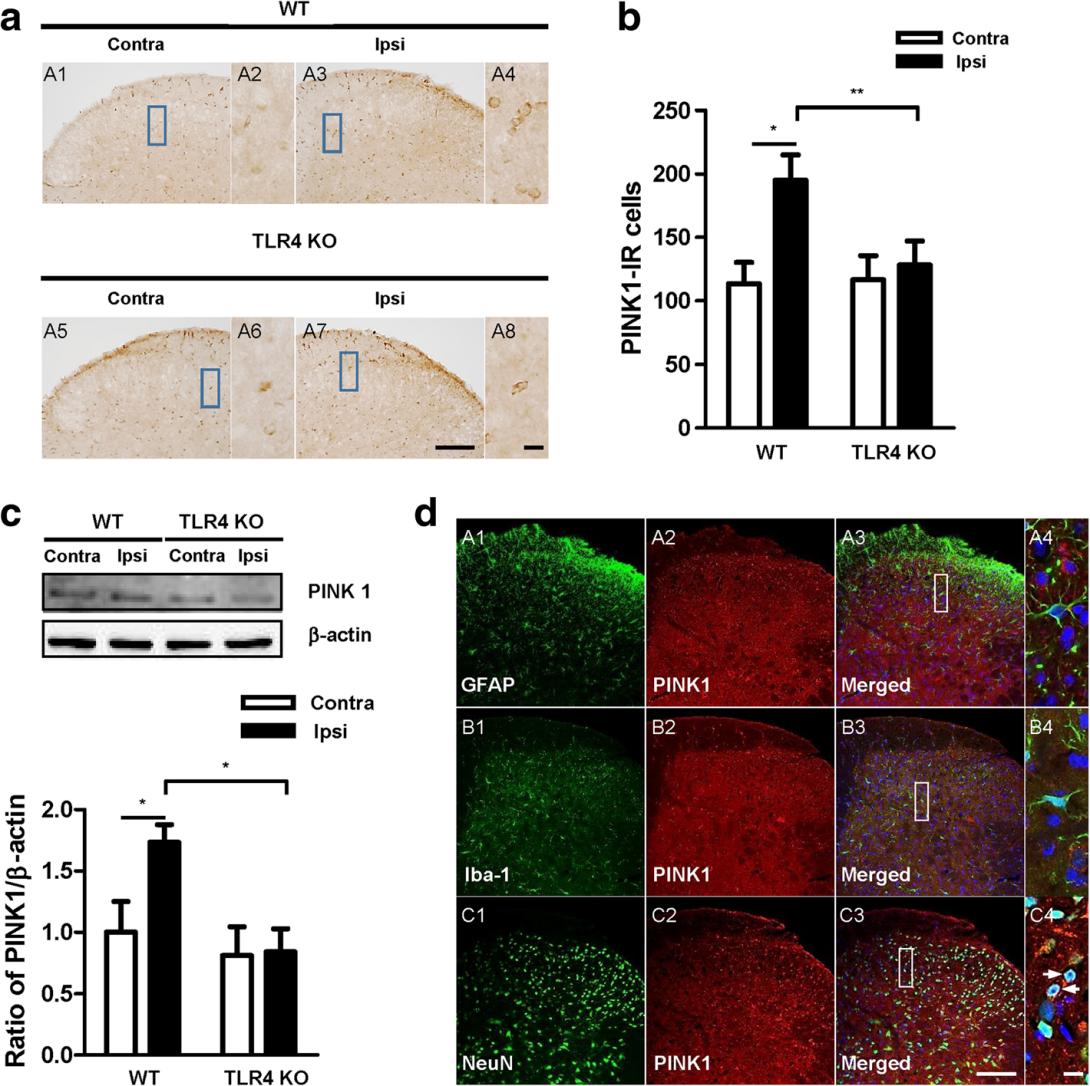
Regulation of the mitophagic marker PINK1 in WT and TLR4 KO mice. **a** PINK1 immunoreactivity was observed in the spinal dorsal horn of CCI mice. PINK1 IR cells were significantly increased in the ipsilateral compared with the contralateral side in WT mice, and no significant difference was found in TLR4 KO mice. Scale bar = 50 μm in A1, A3, A5, and A7. Scale bar = 20 μm in A2, A4, A6, and A8. **b** The number of PINK1 IR cells was significantly increased in the ipsilateral compared with the contralateral side of WT mice. **c** Expression of PINK1 was assessed by Western blotting. The PINK1 protein levels were significantly increased in the ipsilateral side compared with the contralateral side in WT mice, and no significant difference was shown in TLR4 KO mice. (D) Quantification by densitometry with Image J. Two-way ANOVA; all the data are shown as mean ± standard deviation, where *P < 0.05 denotes a significant difference compared with the control group. **d** Frozen sections (WT-CCI, POD7) were stained with PINK1 and co-stained with anti-GFAP (A1–4), anti-iba-1 (B1–4), and anti-NeuN antibodies (C1–4). A4, B4 and C4 is rectangular magnification of merged A3, B3 and C3, respectively. Scale bar = 50 μm in A, B, C1–3. Scale bar = 20 μm in A, B, C4

Mitophagy, the selective degradation of mitochondria by autophagy, often occurs in defective mitochondria following damage or stress [28]. PINK1 is a neuroprotective protein that has been implicated in the activation of mitophagy by selectively accumulating in depolarized mitochondria and promoting PARK2/Parkin translocation [29]. The expression of PINK1 was examined in the spinal cord by immunohistochemical and Western blot analyses. The immunoreactive cells and protein expression of PINK1 were both significantly increased in the ipsilateral side of the spinal dorsal horn and contralateral side in the WT mice, but no significant differences were observed in either side of the spinal dorsal horn of TLR4 KO mice (Fig. 8a, b). Western blot analysis exhibited the same pattern as the immunohistochemical analysis (Fig. 8c). Double immunofluorescence staining to detect the cellular localization of PINK1 showed that PINK1 was expressed in neuronal cells, not astrocyte or microglia in spinal dorsal horn (Fig. 8d).

### Inhibition of autophagy reduced pain behavior

**Fig. 9:**
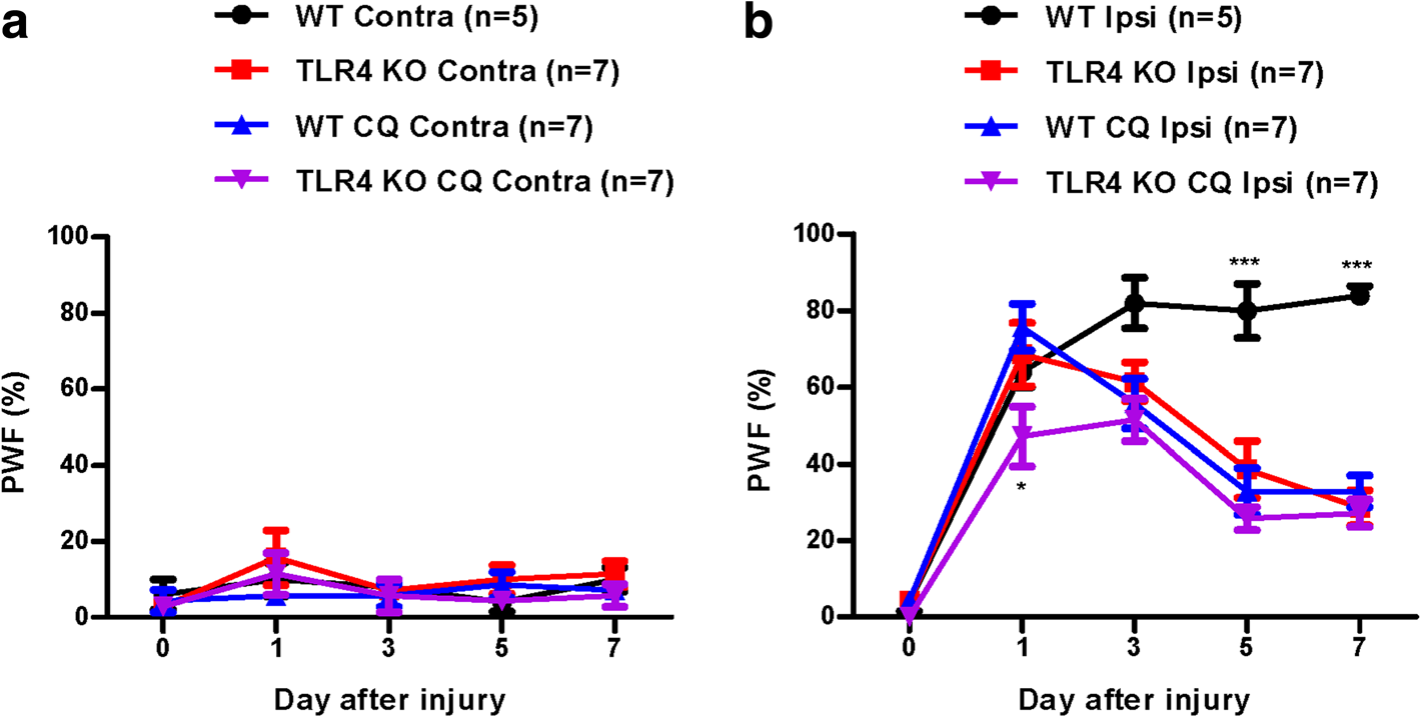
Chloroquine attenuates dramatically mechanical allodynia induced by CCI in WT mice. **a, b** Chloroquine was administrated subcutaneously at 15 mg/kg/day. After 30 min, the paw withdrawal frequency (%PWF) was measured on days 0 (baseline), 1, 3, 5, and 7 after surgery. Mechanical allodynia was separately compared in each group of contralateral (Contra) and ipsilateral (Ipsi) in CCI mice. Two-way analysis of variance (ANOVA); all the data are shown as mean ± SEM, where *P < 0.05 denotes a significant different between TLR4 KO Ipsilateral with TLR4 KO CQ Ipsilateral, ***P < 0.001 WT-CCI ipsilateral vs. the TLR4 KO-CCI ipsilateral

To investigate the role of autophagic flux impairment in the development of neuropathic pain, the autophagic inhibitor, Chlorquine was injected subcutaneously (15 mg/kg/day). After 30 min, the paw withdrawal frequency was measured on days 0 (baseline), 1, 3, 5, and 7 after surgery. Compared with WT ipsi group, Chlorquine treatment significantly reduced the mechanical allodynia at 5 and 7 days in TLR4 KO CQ ipsi group similar with TLR4 KO ipsi group (Fig. 9).

## Discussion

Damage or disease to the somatosensory nervous system that results in disorders of the PNS often leads to chronic neuropathic pain, a debilitating condition resulting from sensitization of the nociceptive pathway. A recent report suggested that the activation of glial cells, especially microglia located in the sensory laminae of the spinal dorsal horn, is the main cause of this process [30]. Activated microglia following peripheral nerve injury changes the morphology and releases neuroactive factors and cytokines that contribute to neuropathic pain [31]. Although the mechanism underlying microglial proliferation following nerve damage remains unclear, it has recently been reported that TLRs play a critical role in neuropathic pain after peripheral nerve injury [7, 32], particularly in microglia activation and driving pain hypersensitivity after nerve injury. TLR4 is an important PAMP and DAMP that regulates the innate or adaptive immune response. TLR4 has been shown to be highly expressed in the CNS of rodents by microglia [10], and genetically altered mice lacking TLR4 showed significantly reduced microglia activation and pain hypersensitivity following nerve injury [7]. Our results showed that the TLR4 KO in mice reduced pain hypersensitivity and proliferation of microglia following CCI-induced nerve injury, verifying the relationship between TLR4 and microglia in neuropathic pain (Fig. 1). CatWalk analysis also showed the correlation between TLR4 and pain hypersensitivity in neuropathic pain. Nerve injury following CCI decreased the percentages of the print area and single stance on the ipsilateral side of mice, and the percentages were significantly increased in TLR4 KO compared with WT mice (Fig. 2).

Autophagy has recently been shown to be a mechanism by which host cells capture and eliminate intracellular pathogens. Pain is a common feature of various neurodegenerative diseases in which autophagy plays a critical role in the progression of the pathology and is being studied as a possible therapeutic target [12, 13]. A recent study demonstrated that autophagy was differently modulated in the spinal cord of mice in several neuropathic pain models [14]. As the most thoroughly characterized type of pattern recognition receptor, TLR4 enhances the elimination of phagocytosed mycobacteria to activate autophagy and serves as an environmental sensor for autophagy. The stimulation of TLR4 with LPS induces autophagosome formation in macrophages by the TRIF-p38 axis and its downstream signaling pathways [15]. Therefore, we hypothesized that the TLR4-mediated autophagy pathway may play a critical role in neuropathic pain. We investigated the spinal modulation of some autophagy markers (e.g., LC3, Beclin 1, and p62) in mice after CCI (Figs. 5–7). In WT mice, increased Beclin 1 levels were paralleled by strong p62 accumulation in the ipsilateral compared with the contralateral side but without a significant increase in LC3-I and LC3-II in both sides of the spinal dorsal horn, suggesting a block in the late phase of autophagic flux rather than an induction of the process. In TLR4 KO mice, however, no significant changes in the three markers were observed in either the ipsilateral or contralateral sides of the spinal dorsal horn, suggesting that the modulation of autophagy was almost blocked due to the lack of TLR4 signaling.

Indeed, Beclin 1 upregulation in WT mice may indicate an increased autophagic flux but also defective autophagosome clearance. In the latter case, Beclin 1 upregulation will be associated with p62 accumulation because this autophagy substrate will not be efficiently degraded by the autophagosomes [23, 33]. Studies on the regulatory role of Beclin 1 in autophagy have suggested that the Beclin 1 complex is involved in autophagosome formation at an early stage [24], and this complex is essential for the recruitment of other autophagy-related proteins to the pre-autophagosomal structure [34].

No significant changes were observed in the expression of LC3-I and LC3-II in both WT and TLR4 KO mice (Fig. 5), and the lipidated form is known to be associated with autophagosomes [22]. Monitoring LC3-II conversion is considered one of the most reliable methods for monitoring autophagy. However, a concomitant increase in both the rate of autophagosome formation and LC3 downstream degradation can show normal steady-state levels in LC3-II despite enhanced autophagy activity [35]. Moreover, LC3 accumulation can result from autophagy induction, but also from impairment at one of the last steps such as fusion with the lysosomes or cargo degradation [33]. Therefore, it is preferable to integrate LC3 studies with the analysis of other components of the autophagic machinery such as members of the initiation complex (e.g., Beclin 1) or autolysosome substrates (i.e., SQSTM1/p62) [23].

One of the best-known autophagic substrates is p62/SQSTM1, a key LC3-binding protein, which serves as a link between LC3 and ubiquitinated proteins [27]. p62 and p62-bound polyubiquitinated proteins become incorporated into the completed autophagosome and are degraded in autolysosomes. Because of the correlation between autophagy modulation and p62 levels [27, 36, 37], this substrate is considered a useful readout of autophagic degradation [23, 38]. Indeed, p62 levels increase when autophagy is impaired [37]. In WT mice, ipsilateral p62 accumulation was observed to significantly increase compared with the contralateral side, suggesting a block in the final degradative steps of autophagy. However, in the TLR4 KO mice, no significant change in the p62 level was observed between the two sides of the spinal dorsal horn (Fig. 7). Altogether, the analysis of LC3, Beclin 1, and p62 in this study indicated that autophagy impairment in CCI-induced neuropathic pain may be due to the occurrence of a block in the late phase of autophagic flux rather than in the induction of the process, and this autophagy seems to be mediated by TLR4 signaling. Moreover, mitophagy was assessed by monitoring the expression of the protein marker PINK1 (Fig. 8). Expression of PINK1 was increased in WT mice after CCI but showed no significant change in TLR4 KO mice, indirectly supporting our hypothesis regarding TLR4-mediated autophagy.

Although it was early reported that TLR4 is expressed primarily in microglia, but not astrocytes or neurons [39], it was also found that neurons do express TLR4 and that TLR signaling in neurons regulates neural precursor cell proliferation axonal growth, adult neurogenesis, and neuronal plasticity [40]. In this study, we found that the expression of LC3, Beclin 1, and p62 associated with autophagy impairment in CCI-induced neuropathic pain was localized with neuronal cell, not astrocyte or microglia, in spinal dorsal horn. Previously, Tanga et al., reported that the genetically altered mice displayed significantly attenuated behavioral hypersensitivity and decreased expression of spinal microglial markers and proinflammatory cytokine [7]. Therefore, it is reasonable that TLR4-mediated pro-inflammatory cytokine release in microglia and TLR4-mediated autophagic impairment in neurons contribute pain sensory hypersensitivity synergically.

Our immunohistochemical studies were supported with autophagic inhibitor, Chlorquine treatment. Chloroquine (Sigma-Aldrich, St. Louis, MO) is one of many compounds which have shown to reverse autophagy by accumulating in lysosomes, disturbing the vacuolar H^+^ ATPase, which is responsible for lysosomal acidification and blocking autophagy [41]. When injected intrathecally in WT, Chloroquine induced a significant reduction in threshold of mechanical sensitivity (Fig. 9b). This data is in perfect agreement with previous paper [14]. In that paper, the authors showed chloroquine was able to modulate the spinal autophagic machinery by the increase in p62, indicative of autophagosome accumulation. However, they did not show data on comparison with TLR4 KO mice. Our data showed that CCI-induced mechanical allodynia in TLR4 KO with Chloroquine treatment attenuated pain threshold compared to TLR4 KO. This data strongly supported TLR4-mediated autophagic impairment in neurons contribute pain sensory hypersensitivity with microglia activation, whereas, microglial TLR4-mediated microglial activation might be indirectly coupled to autophage.

**Fig. 10:**
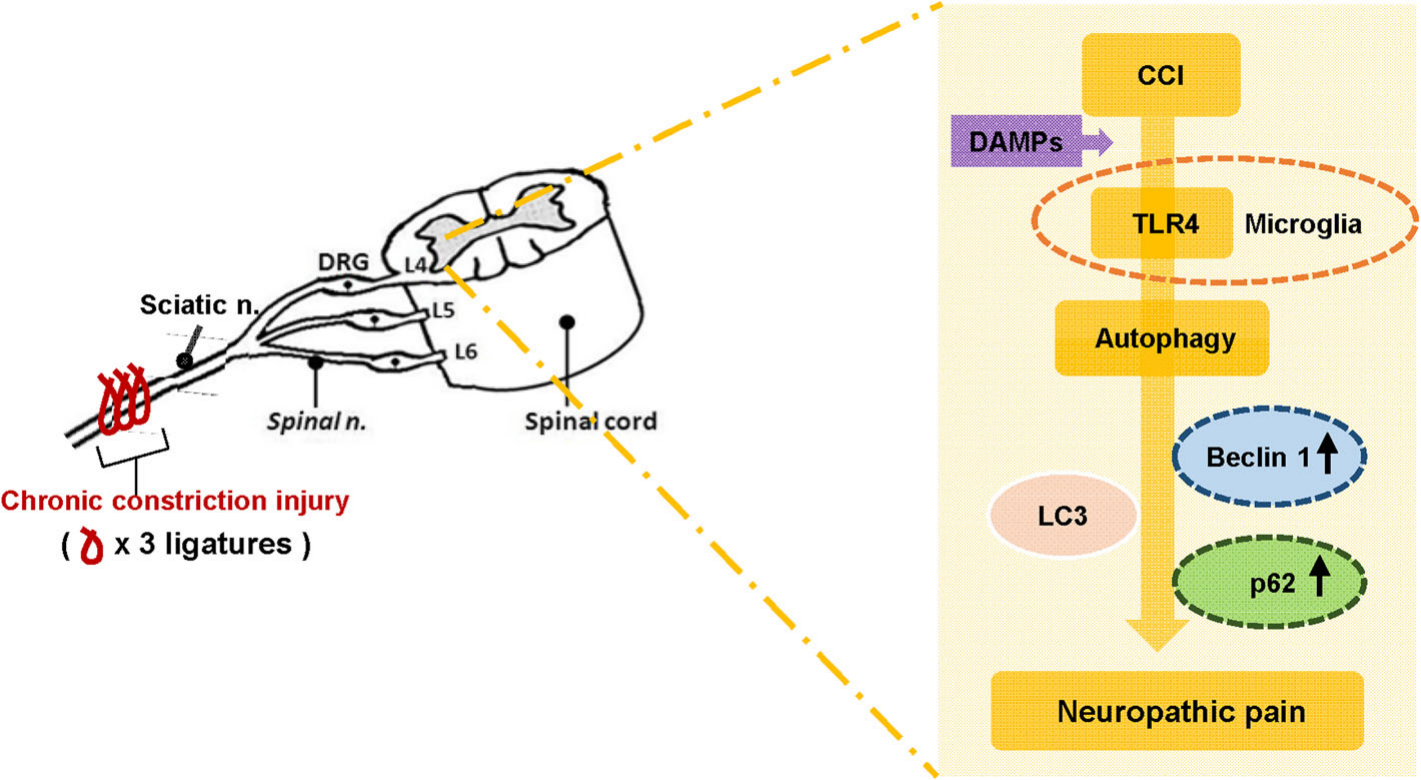
CCI-induced nerve injury leads to neuropathic pain by diverse molecular mechanisms. The CCI-induced neuropathic pain mouse model, in which ligatures are placed loosely around the right common sciatic nerve at the mid-thigh level, causes nerve injury and leads to the release of endogenous TLR4 agonists (such as pathogen-associated molecular patterns [PAMPs] and damage-associated molecular patterns [DAMPs]) in the spinal cord. These agonists activate spinal cord microglia and astrocytes through TLR4, which can lead to increased neuronal activation of autophagy, resulting in the increased regulation of autophagic proteins (Beclin 1, p62) and ultimately leading to neuropathic pain

In conclusion, the present study demonstrated that the deficiency of glial TLR4 could decrease mechanical allodynia with synergic TLR4 mediated blockade of impaired spinal autophagy induction in CCI-induced neuropathic pain mice (Fig. 10). Additionally, our study improves our understanding of TLR4 autophagy-related neuropathic pain and provides the scientific basis for the use of TLR4 and autophagy as potential therapeutic targets in the clinical management of neuropathic pain.

## Methods

### Experimental animals

Eight-week-old male C57BL/6j mice (Narabiotech, Seoul, Korea) were used in this study. C57BL/10ScNJ TLR4-KO (TLR4^−/−^) mice were purchased from the Jackson Laboratories (Bar Harbor, ME, USA). All the mice were individually housed in cages on a standard 12 h/12 h light/dark cycle, and water and food were available ad libitum.

### Mechanical allodynia assay

To assess the sensitization to innocuous mechanical stimulation (mechanical allodynia), we measured the paw withdrawal response frequency (PWF) using a von Frey filament (North Coast Medical, Morgan Hill, CA, USA) as described in a previous study [42]. Based on that study, a von Frey filament with a force of 2.0 g was selected for testing. Mice were placed on a metal mesh flooring, and the von Frey filament was applied from underneath the metal mesh flooring to each plantar of the hind paw. The filament was applied 10 times to each paw at intervals of 10 s, and the number of paw withdrawal responses following each filament stimulus was counted. The result of each experimental animal was expressed as a percentage of the paw withdrawal response frequency (% PWF). Paw withdrawal responses were measured day 0 (baseline), 1, 3, 5, and 7 after CCI surgery in each set.

### CCI-induced neuropathic pain

CCI of the common sciatic nerve was performed based on the method described by Bennett and Xie [18]. Mice were anesthetized with an intraperitoneal injection (i.p.) of Avertin (2,2,2-tribromoethanol, 50% *w*/*v* in tertiary amyl alcohol, diluted 1:40 in H_2_O; 20 ml/kg, i.p.; Sigma-Aldrich, St. Louis, MO, USA). The right common sciatic nerve was exposed at the mid-thigh level and was dissected from the connective tissue. Three loose ligatures of the 4–0 chromic gut were tied around the nerve with an interval of 1.0 to 1.5 mm between each ligature. After surgery, mice recovered on the heating pad at 27 °C.

### CatWalk-automated gait analysis

The CatWalk XT system (Noldus Information Technology, Wageningen, The Netherlands) was used for the quantitative assessment of the gait parameter and footfalls in rodents. CatWalk is a verified system in the research of several pain models such as spinal cord injury, traumatic brain injury, and neuropathic pain. During the test, the mice traversed a dark tunnel with a glass plate from one side to the other. Their footprints were illuminated by fluorescent light from the glass plate and were captured by a high-speed camera positioned underneath the plate. The captured images were immediately processed by CatWalk XT software, and numerous parameters were analyzed, such as the print area, swing speed, and single stance. In this study, we measured the print area and single stance to assess differences in nociceptive responses between WT and TLR4-KO CCI mice. The print area is the contacting area between the hind paw and glass, and a single stance is the duration of the contralateral or ipsilateral hind paw touching the glass plate in the step cycle. CatWalk gait analysis was measured before and 1, 3, 5, and 7 days after CCI surgery in each set.

### Immunostaining analysis

Immunohistochemistry was performed 7 days after surgery. The mice were anesthetized with sodium pentobarbital (50 mg/kg, i.p.) and perfused transcardially with heparinized phosphate-buffered saline (PBS, pH 7.4), followed by perfusion with 4% paraformaldehyde for 15 min. The lumbar enlargement (L4–L6) regions of the spinal cords were removed immediately, immersed in the same fixative overnight, and embedded in paraffin. The paraffin-embedded tissue arrays were performed in 4-μm sections and deparaffinized and rehydrated in a graded alcohol solution. The sections were soaked in 0.01 M citrate buffer (pH 6.0) and heated in a microwave vacuum histoprocessor (RHS-1, Milestone, Bergamo, Italy) at a controlled final temperature of 121 °C for 15 min for antigen retrieval. For immunohistochemical analyses as previously [43], endogenous peroxidase activity was blocked using 0.3% hydrogen peroxide. After primary antibody reaction (4 °C, overnight) as follows; Becline1(1:400; #AP1818a, ABGENT), p62 (1:400; #p0067, Sigma-Aldrich), PINK1 (1:400; #NBP2–36488, Novus Biologicals, Littleton, CO, USA), LC3 (1:200; #sc376404, Santa Cruz Biotechnology, Santa Cruz, CA, USA), NeuN (1:200; #24307S, Bioncompare), NeuN (1:200; #MAB377, Millpore), GFAP (1:2000, #Z0334, Dako), GFAP (1:2000; #MAB360, Millpore), Iba-1 (1:400; #019–19,741, Wako), Iba-1 (1:400; #016–26,721, Wako), the tissues were exposed to biotinylated anti-rabbit IgG and streptavidin peroxidase complex (Vector Laboratories, Inc., Burlingame, CA, USA). Immunostaining was visualized with diaminobenzidine (DAB), and the specimens were mounted using Polymount (Polysciences, Inc., Warrington, PA, USA).

### Western blot analysis

The lumbar enlargement (L4-L6) regions of the spinal cord from WT and TLR4 KO mice were dissected and homogenized in lysis buffer. The lysates of the spinal cord (20 μg) were separated by 12 or 15% sodium dodecyl sulfate-polyacrylamide gel electrophoresis (SDS-PAGE) and transferred to nitrocellulose membranes. The blots were probed with the following primary antibodies: Becline 1 (1:1000; #sc-11,427, Santa Cruz Biotechnology, Santa Cruz, CA, USA), LC3 (1:1000; #L8918, Sigma-Aldrich), p62 (1:1000; #P0067, Sigma-Aldrich), PINK1 (1:1000; #NBP1–39667, Novus Biologicals, Littleton, CO, USA), and β-actin (1:500; #2965, Cell Signaling Technology, Danvers, MA, USA). The immune complexes were identified using an enhanced chemiluminescence (ECL) detection system (Habersham, Little Chalfont, United Kingdom).

### Statistical analysis

All the data are presented as mean ± standard error of the mean. Quantitative analysis of immunostaining was performed using ImageJ (National Institutes of Health, Bethesda, MD, USA) as previously [43]. Statistical analyses were performed using the statistical software Prism 6.0 program (Graph Pad Software, San Diego, CA, USA), and repeated measurements from behavioral studies were analyzed by two-way analysis of variance. The results were considered significant at *P < 0.05, **P < 0.01, and ***P < 0.001.
